# The Operative Treatment of Empyena and Abscess of the Lung

**Published:** 1892-07-23

**Authors:** 


### SURGERY.

### THE OPERATIVE TREATMENT OF EMPYEMA AND ABSCESS OF THE LUNG.

When any important operation is introduced it is sure to be followed before long by some modification which requires a new name to distinguish it from the original; consequently it is important, in dealing with the subject of this paper, to be sure, at the outset, of our nomenclature. The essential element in these operations is the opening in the chest wall, whether we wish to remove pus from the pleural cavity or from the lung itself, or to remove diseased pulmonary or pleural tissue. It seems unnecessary, therefore, to differentiate too sharply; what we desire is some terms which will indicate, chiefly, the treatment of the chest wall. The word "Pleurotomy" (πλευρὰ—τέμνω), sometimes used, indicates an opening into the pleura, but it might also mean, from its derivation, any cutting operation on the chest wall, or ribs. As "Thoracentesis" has become a recognised name for tapping the thorax, it would seem desirable to use other terms formed on this pattern. Two such are employed by English surgeons, viz., *Thoracotomy* (θώραξ—τέμνω), meaning either a simple incision into the thoracic wall, or the removal of a portion of rib in order to increase the size of the opening. Secondly, we have the somewhat inharmonious word *Thoracoplasty* (θώραξ—πλαστὸς), which signifies taking away some part of the chest wall (portions of two or three ribs, perhaps), in order to freely expose a cavity which has remained for some time. The latter is also called the operation of Esthlander. These two may be considered separately.

(1) *Thoracotomy.* This operation, as described by Godlee, Treves, Huber, &c., is as follows: We will suppose that the temperature, localised dulness, and other signs, point to a collection of pus in the pleural cavity. The history of the case is important, because it is well to know, if possible, whether the lung is adherent to the parietes or not, also if the disease originates from a pulmonary cavity breaking into the pleura, &c.

Dr. Rickards (*British Medical Journal*, May 21st, 1892) urges the necessity of early interference in cases of empyema, and the advantages of a free incision.

The site of the skin wound must, of course, depend on circumstances, but if permissible, Mr. Godlee and others recommend the eighth or ninth space, just external to the angle of the scapula. The opening must not be oblique and valvular, and to avoid this it is better to make a mark on the skin at the spot chosen before the operation; otherwise, during the manipulation of the arm, &c., the superficial and deep wounds may not correspond.

The incision should be three or four inches in length and in the course of the ribs. Should there be any doubt about the position of the pus, an exploring needle or trocar should be introduced, and the track of this needle followed up and opened with a sinus forceps or scalpel. Mr. Agnew considers the entrance of air of little consequence; many surgeons agree with him and make no special precautions to avoid it. Dr. Rickards, however, maintains (*op. cit.*) that it is most important to keep air out of the wound to ensure the subsequent expansion of the lungs; and he points out that if this expansion does not take place the ultimate result is almost always bad, although there may be a temporary improvement. He recommends that a piece of oil-silk eight inches square be quickly applied to the wound whilst the pus is still flowing from it; this acts as a moist valve, and prevents air from entering. A drainage tube (with or without a counter opening) should be inserted; it should be as short as is consistent with good drainage. The wound should be dressed with abundance of absorbent, aseptic material, and the next day it is advisable to wash out the opened cavity with a weak corrosive sublimate solution (1 in 8,000) at a temperature of 100 deg. F.

In some cases of empyema, in abscess of the lung, &c., it may be necessary to resect a portion of rib in order to ensure a better opening. It is best to strip off the periosteum carefully, in order to avoid injury to the intercostal vessels, for bleeding from this source would very much interfere with the operation at this stage. The rib or ribs are sawn through with a fine saw, and removed with a forceps to the required extent. The periosteum, with the vessels, is then cut through and removed, the arteries being ligatured or twisted. Usually a length of one or two inches of bone is removed.

*Thoracoplasty.*—The object of this is to remove the whole thoracic boundary of some chronic cavity in the pleura; it is a more severe operation than the last, but it resembles it in many particulars. The skin incision must be so arranged as to allow freedom in manipulating the ribs. Esthlander himself employs one or more transverse cuts in the direction of the ribs; Gould recommends a vertical, and Godlee a U-shaped incision. Delagenière has recorded twenty cases of this nature, and reports one in *La Semaine Médicale* for April 30th, 1892, where an abscess of the lung was diagnosed, and the seventh, eighth, and ninth ribs were resected periosteally from their angles to their junction with the cartilages. The gangrenous lung was removed with a scissors, and he advocates the removal of diseased tissue, whether pulmonary or pleural, and not merely the evacuation of pus. As might be expected, there is considerable shock, but good results have followed in several cases.

As regards abscess of the lung, it should be noted that there is nothing but the aspirating needle that can make the diagnosis absolutely certain. In his articles in the *Lancet* in March, 1887, Mr. Godlee describes the operation for evacuating the contents, which should be done in the way already described (Thoracoplasty). The after treatment consists in syringing out with carbolic lotion. As a rule this should not be done at the time of the operation, and if troublesome coughing is set up it should be abandoned for a time, or the strength of the solution lessened. If irritation is easily produced, a boracic acid solution or merely sterilized warm water may be used.

A complication occurs if the lung is not adherent to the chest wall, for the escape of pus and air into the pleural cavity becomes probable in such cases. The only thing to be done is to fasten the pleura to the parietes round the edges of the opening. Mr. Godlee describes this as a somewhat difficult proceeding. Huber has recently reported an operation of this nature which was followed by an attack of acute pneumonia, but the patient ultimately got well.

